# Effect of early endothelial function improvement on subclinical target organ damage in hypertensives

**DOI:** 10.1038/s41598-024-67143-1

**Published:** 2024-07-12

**Authors:** Xiaodong Huang, Xianwei Huang, Mandong Pan, Jiyan Lin, Liangdi Xie

**Affiliations:** 1 Department of Emergency, The First Affiliated Hospital of Xiamen University, School of Medicine, Xiamen University, Xiamen, 361003 China; 2 Xiamen Key Laboratory for Clinical Efficacy and Evidence-Based Research of Traditional Chinese Medicine, The First Affiliated Hospital of Xiamen University, Xiamen, 361003 China; 3 Department of Geriatrics, Fujian Hypertension Research Institute, The First Affiliated Hospital of Fujian Medical University, 20 Chazhong Road, Fuzhou, 350005 China

**Keywords:** Essential hypertension, Subclinical target organ damage, Endothelial function, Flow-mediated dilation, Cardiology, Diseases, Medical research

## Abstract

Endothelial dysfunction is acknowledged as a marker for subclinical target organ damage (STOD) in hypertension, though its therapeutic potential has not yet been clarified. This study assessed whether early endothelial function improvement (EEFI) reduced STOD in patients with essential hypertension (EH). We conducted a retrospective cohort analysis of 456 EH patients initially free from STOD. Endothelial function was assessed using brachial artery flow-mediated dilation (FMD), with values ≤ 7.1% indicating dysfunction. Patients were initially categorized by endothelial status (dysfunction: n = 180, normal: n = 276), and further divided into improved or unimproved groups based on changes within three months post-enrollment. During a median follow-up of 25 months, 177 patients developed STOD. The incidence of STOD was significantly higher in patients with initial dysfunction compared to those with normal function. Kaplan–Meier analysis indicated that the improved group had a lower cumulative incidence of STOD compared to the unimproved group (*p* < 0.05). Multivariable Cox regression confirmed EEFI as an independent protective factor against STOD in EH patients (*p* < 0.05), regardless of their baseline endothelial status, especially in those under 65 years old, non-smokers, and with low-density lipoprotein cholesterol levels ≤ 3.4 mmol/L. In conclusion, EEFI significantly reduces STOD incidence in EH patients, particularly in specific subgroups, emphasizing the need for early intervention in endothelial function to prevent STOD.

## Introduction

Hypertension affects roughly one billion individuals worldwide, making it a prevalent chronic disease^1^. As a major risk factor for cardiovascular diseases, it poses a threat to public health globally^2^. Current treatment strategies focus on comprehensive risk management beyond mere blood pressure control, emphasizing target organ protection and clinical event reduction^3^. Endothelial dysfunction is crucial in hypertension development, serving as both an early biomarker of elevated blood pressure and a warning for future cardiovascular events^4,5^. However, limited evidence exists on how early endothelial function improvement (EEFI) can prevent the progression of subclinical target organ damage (STOD) in hypertensive patients.

Flow-mediated dilation (FMD), a non-invasive technique, assesses endothelial function and accurately reflects vascular health. Using this technology, our prior research^6–10^ has investigated the link between endothelial function and hypertension, demonstrating that endothelial dysfunction correlates with a heightened risk of progression and complications in essential hypertension (EH). Based on these findings, we hypothesized that EEFI would reduce the incidence of STOD in EH patients. We aimed to explore this hypothesis through empirical research. This study sought to explore the potential of EEFI to prevent STOD in EH patients. The findings could enhance the understanding of EH progression and inform prevention and treatment strategies for hypertension.

## Methods

### Experimental protocol

This single-center retrospective cohort study drew from the *Hypertension Target Organ Damage and Its Risk Factors—Fuzhou Study* database. Ethical clearance was granted by the Ethics Committee of the First Affiliated Hospital of Fujian Medical University (Ref: MRCTA, ECFAH of FMU (2020) 306). Additionally, the study was registered with the China Clinical Trial Registry under registration number ChiCTR2000039448 (Dated: 28/10/2020), available at http://www.chictr.org.cn/index.aspx. The study conformed to the Declaration of Helsinki guidelines, with all participants having read and signed informed consent forms before participating.

### Participants

This study analyzed EH patients from the First Affiliated Hospital of Fujian Medical University, with follow-up data spanning August 1, 2000, to May 31, 2016. Hypertension diagnosis followed the 2018 Chinese Guidelines for Prevention and Treatment of Hypertension^11^. STOD diagnostic criteria: (1) cardiac: echocardiography showing left ventricular mass index (LVMI) ≥ 115 g/m^2^ for males or ≥ 95 g/m^2^ for females; (2) renal: mildly elevated serum creatinine (males 115–133 μmol/L, females 107–124 μmol/L), reduced glomerular filtration rate [30–59 mL/(min·1.73 m^2^)], proteinuria-to-creatinine ratio ≥ 30 mg/g; (3) vascular: carotid intima-media thickness (CIMT) ≥ 0.9 mm or arterial plaques. Endothelial function improvement was defined as a return to normal for initially dysfunctional patients, and an enhancement beyond baseline for those initially normal, after three months of treatment. Inclusion criteria: (1) confirmed EH; (2) no evidence of STOD or any cardiovascular disease (e.g., myocardial infarction, stroke, or peripheral artery disease). Exclusion criteria were: secondary hypertension, presence of STOD or cardiovascular disease, heart failure, severe arrhythmias, hematologic or rheumatologic conditions, significant liver or kidney dysfunction, thyroid issues, cancer, recent serious infections, or immunosuppressant use^7,8^.

**Figure 1:**
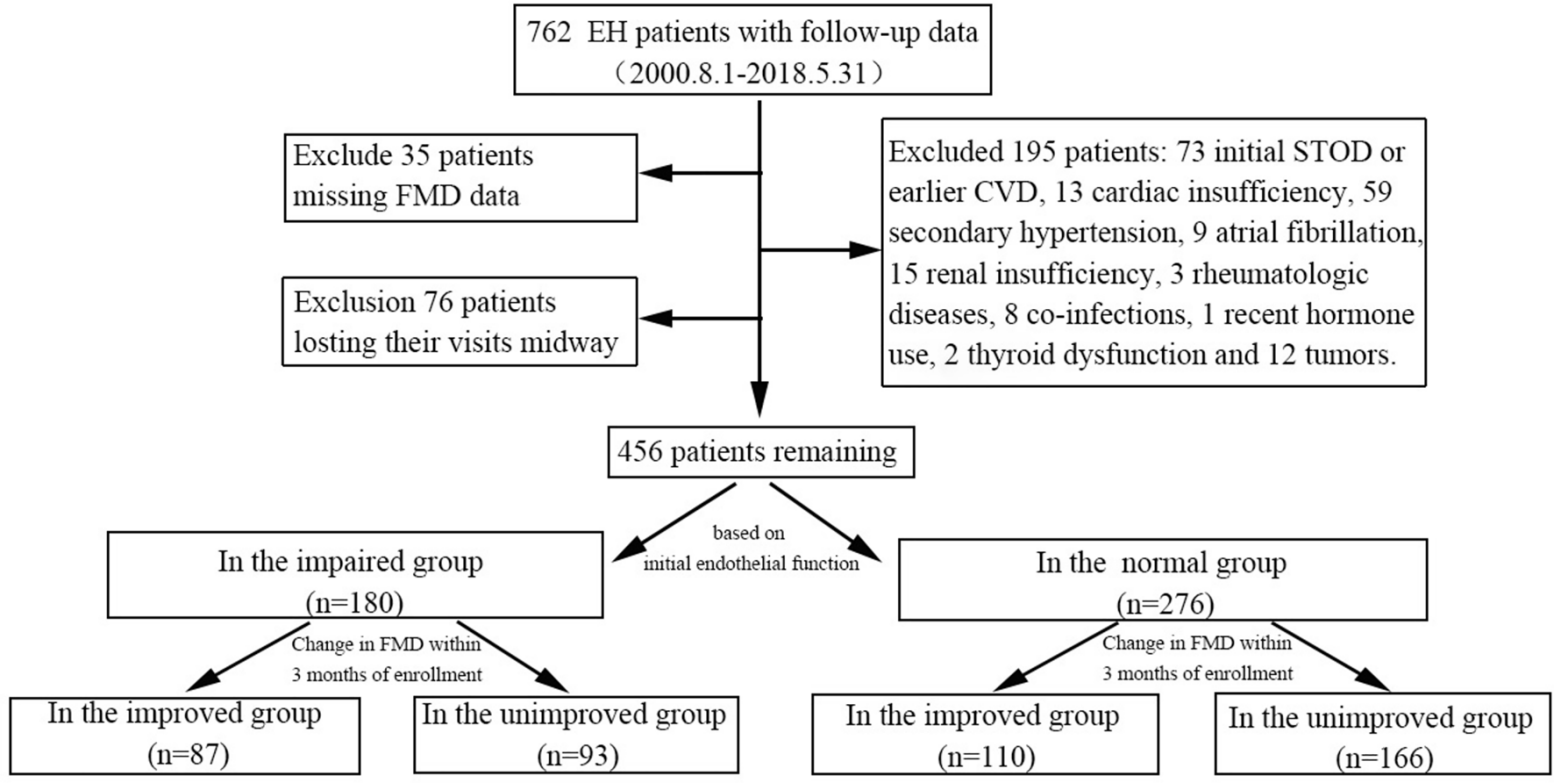
Flowchart for identifying study cohorts from the *Hypertension Target Organ Damage and Its Risk Factors—Fuzhou Study* database. *EH* essential hypertension, *FMD* flow-mediated dilation, *CVD* cardiovascular disease.

All patients received treatment as per the standards outlined in the Chinese guidelines for hypertension management^11^. Follow-up was conducted every 1 to 3 months by telephone appointment, encompassing both outpatient and inpatient visits. If patients did not comply, certain target organ assessments (e.g., CIMT) were postponed to a 6-month interval. The follow-up endpoint was the occurrence of STOD, with the interval from enrollment to STOD onset was defined as the survival period. The follow-up cut-off date was May 31, 2018, with a median follow-up time of 25 months (95%CI 10–63). Initially intended to follow 762 patients, the study ultimately included 456 due to exclusion criteria, losses to follow-up, and missing FMD data. Patients were categorized into groups based on initial endothelial function: impaired and normal. Within three months of enrollment, they were further subdivided into improved and unimproved groups based on improvement in endothelial function (Fig. 1).

### Clinical data collection

Upon admission, data on participants’ age, gender, drinking and smoking habits, duration of hypertension and medical history were collected. Current smokers were defined as individuals who smoked at least one cigarette daily for over six months. Drinkers were defined as males consuming over 25 g and females over 15 g of alcohol per day^10^. Participants were required to abstain from coffee and strong tea for half an hour before the examination. Following a 5-min rest, blood pressure and heart rate were measured with a HEM-7052 automatic sphygmomanometer (Omron, Japan), averaging three readings^10^. Height and weight were measured, and then body mass index (BMI) was calculated as weight (kg) divided by the square of height (m^2^). Blood samples were drawn from the antecubital vein of all subjects in the morning after an 8-h fast. Subsequently, the samples were analyzed for total bilirubin, alanine aminotransferase, lactate dehydrogenase, uric acid, creatinine, total cholesterol (TC), high-density lipoprotein-cholesterol, low-density lipoprotein-cholesterol (LDL-C), and fasting serum glucose (FSG) using an ADVIA 2400 automatic analyzer (Siemens, Germany)^10^. Urinary albumin was assessed by immunoturbidimetry (Roche P800, USA), and urinary creatinine by colorimetric method (Boehringer Mannheim/Hitachi 717 system, Germany), to calculate the urine albumin-to-creatinine ratio (UACR)^10^. The estimated glomerular filtration rate (eGFR) was calculated using the Chronic Kidney Disease Epidemiology Collaboration equation^12^.

### Flow-mediated dilation

This study adhered to international ultrasound guidelines for vascular function^13^ and integrated previous research^6–10^ to establish a standardized testing protocol. Patients were positioned supinely, with the target vessel, the brachial artery segment 5 cm above the right elbow crease, clearly marked. Following a 10-min rest, the basal inner diameter of the brachial artery was measured three times using a LOGIQ7 color doppler ultrasound (GE, USA), and the average was recorded as D_0_. A cuff was placed 5–17.5 cm above the elbow, inflated to 200 mmHg or 50 mmHg above systolic pressure, obstructing blood flow for 5 min, followed by rapid deflation. After 80–90 s of decompression, the diameter of the target vessel (D_1_) was measured^6–10^. Endothelial-dependent vascular function was calculated using the formula FMD = (D_1_ − D_0_) × 100%/D_0_, defining FMD ≤ 7.1% as endothelial dysfunction^6,7,14^. Vascular function was assessed by three researchers trained in the same standardized method, with an intra-operator coefficient of variation of 1.9% and an inter-operator coefficient of variation of 6.92%^6–10^.

### Carotid ultrasound and echocardiography

The examination was performed using a LOGIQ7 color Doppler ultrasound (GE, USA) by an experienced physician unaware of the clinical details. During the carotid ultrasound, participants lay flat, without a pillow, turning their head towards the examined side. The ultrasound continuously scanned from the right brachiocephalic artery’s bifurcation to the left aortic arch’s origin, covering both carotid arteries and their branches. CIMT was measured five times 1.5 cm proximal to the common carotid artery bifurcation, and the average was taken as the result^15^. If plaques were present, measurements were taken at the same proximal location. During echocardiography, the physician measured left ventricular end-diastolic diameter (EDD), septal wall thickness (SWT), and posterior wall thickness (PWT) using M-mode tracing while participants rested. Left ventricular mass (LVM) was calculated with the formula: 0.8 × 1.04 × [(EDD + SWT + PWT)^3^ − EDD^3^] + 0.6. Body surface area (BSA) was calculated from height and weight, and LVMI was derived by dividing LVM by BSA^16^.

### Statistical analyses

Data processing and analysis utilized SPSS version 22.0. Tests for normal distribution and homogeneity of variance preceded analysis of continuous variables. Normally distributed data were reported as mean ± standard deviation, while non-normally distributed data were reported as median and interquartile distance. Categorical data were expressed as frequencies (percentages). Group differences were assessed using Pearson or Fisher's chi-square tests, Student's t-test, and Mann–Whitney *U* test. Kaplan–Meier survival curves and cumulative hazard curve for the occurrence of outcome events in the two groups of patients were plotted using the "survival + survminer" package in R software (version 4.2.1), with group differences compared using the log-rank test. Finally, multivariate Cox regression analysis determined the effects of EEFI on STOD and identified additional potential risk factors. Subgroup analysis was performed, with results visualized using the "forestploter" package. Statistical significance was defined as a two-sided *p*-value < 0.05.

## Results

### Comparison of patient baseline characteristics

This study included 456 patients with EH, 180 with endothelial dysfunction, and 276 with normal endothelial function. Eventually, 177 patients developed STOD. All patients included in the study were free from any previous cardiovascular diseases at baseline. Compared to those with normal function, the endothelial dysfunction group featured a higher proportion of men, smokers, and individuals with diabetes, and displayed increased age as well as extended duration of EH (*p* < 0.05). Additionally, this group had lower levels of FMD, but higher uric acid levels (*p* < 0.05). In terms of medication, patients with initial endothelial impairment used more statins and fewer β-blockers than those with normal function (*p* < 0.05). No significant differences were observed in other baseline data between the two groups (Table 1).

**Table 1:** Clinical characteristics of hypertensives patients with and without initial endothelial impairment.

Variables	ALL(n = 456)	Impaired group (n = 180)	Normal group (n = 276)	*P* value
Female, n (%)	214 (46.93%)	66 (36.67%)	148 (53.62%)	0.001
Age (years)	61.11 ± 11.67	62.65 ± 10.96	60.11 ± 12.03	0.023
Current smoking [n (%)]	135 (29.61%)	71 (39.44%)	64 (23.19%)	< 0.001
Drinking [n (%)]	53 (11.62%)	23 (12.78%)	30 (10.87%)	0.534
Duration of EH (months)	3 (0–10)	4 (1–10)	2 (0–8)	0.001
Diabetes mellitus [n (%)]	58 (12.72%)	34 (18.89%)	24 (9.06%)	0.002
BMI (kg/m^2^)	24.25 ± 3.02	24.59 ± 2.72	24.03 ± 3.18	0.053
SBP (mmHg)	151.04 ± 11.13	151.82 ± 12.54	150.53 ± 10.10	0.248
DBP (mmHg)	85.05 ± 12.05	85.13 ± 12.29	85.00 ± 11.90	0.908
Heart rate (bp)	72.13 ± 9.07	71.59 ± 9.78	72.47 ± 8.58	0.314
TBil (μmol/L)	14.40 ± 6.26	14.75 ± 6.64	14.18 ± 6.00	0.355
ALT (U/L)	24 (17–34)	24 (18–35)	23 (17–33)	0.120
LDH (U/L)	177.26 ± 45.61	181.13 ± 54.81	174.81 ± 37.57	0.164
Uric acid (μmol/L)	349.03 ± 89.85	361.12 ± 96.59	340.99 ± 84.30	0.028
eGFR (mL/min/1.73m^2^)	89.48 ± 14.83	87.38 ± 14.28	90.87 ± 15.05	0.018
Total cholesterol (mmol/L)	4.99 ± 1.10	4.94 ± 1.04	5.02 ± 1.13	0.458
HDL-C (mmol/L)	1.37 ± 0.40	1.38 ± 0.40	1.37 ± 0.40	0.896
LDL-C (mmol/L)	3.04 ± 0.97	2.94 ± 0.93	3.11 ± 0.98	0.074
FSG (mmol/L)	5.77 ± 1.26	5.91 ± 1.34	5.68 ± 1.20	0.065
CIMT (cm)	0.06 ± 0.01	0.06 ± 0.01	0.06 ± 0.01	0.075
UACR (mg/g)	6.99 (3.82–13.10)	6.61 (3.33–13.90)	7.47 (4.10–13.05)	0.419
LVMI (g/m^2^)	81.61 ± 19.54	84.80 ± 23.23	79.91 ± 17.06	0.047
ACEI/ARBs [n (%)]	237 (51.97%)	92 (51.11%)	145 (52.54%)	0.766
β-blockers [n (%)]	116 (25.44%)	33 (18.33%)	83 (27.07%)	0.005
Calcium channel blockers [n (%)]	235 (51.54%)	97 (53.89%)	138 (50.00%)	0.417
Diuretics [n (%)]	145 (31.80%)	65 (36.11%)	80 (28.99%)	0.110
Statin [n (%)]	169 (37.06%)	91 (50.56%)	78 (28.26%)	< 0.001
FMD (%)	9.40 ± 6.57	3.75 ± 3.01	13.08 ± 5.58	< 0.001

Data are expressed as means ± SD or median (25th–75th).

*EH* essential hypertension, *BMI* body mass index, *SBP* systolic blood pressure, *DBP* diastolic blood pressure, *TBil* total bilirubin, *ALT* alanine aminotransferase, *LDH* lactate dehydrogenase, *HDL-C* high-density lipoprotein-cholesterol, *LDL-C* low-density lipoprotein-cholesterol, *FSG* fasting serum glucose, *CIMT* carotid intima-media thickness, *UACR* urinary albumin to creatinine ratio, *LVMI* left ventricular mass index, *ACEI* angiotensin-converting enzyme inhibitor, *ARB* angiotensin receptor blocker, *FMD* flow-mediated dilation.

Patients were categorized based on initial endothelial function for further analysis. In the impaired group, the improved subgroup had significantly fewer smokers than the unimproved subgroup (*p* < 0.05), with similar other baseline characteristics. Despite similar blood pressure control, the improved subgroup used more renin-angiotensin system (RAS) inhibitors and fewer diuretics than the unimproved subgroup (Table 2). In the normal group, those in the improved subgroup were younger with higher eGFR levels than the unimproved subgroup (*p* < 0.05). There were no significant abnormalities between the groups in terms of initial treatment medications and other baseline data (Table 3).

**Table 2:** Clinical characteristics of patients with initial endothelial impairment who improved vs. those who did not improve after 3 months of enrollment treatment.

Variables	Improved group (n = 87)	Unimproved group (n = 93)	*P* value
Female, n (%)	31 (35.63%)	35 (37.63%)	0.781
Age (years)	62.99 ± 10.16	62.3 ± 11.7	0.973
Current smoking [n (%)]	25 (28.74%)	46 (49.46%)	0.004
Drinking [n (%)]	8 (9.20%)	15 (16.13%)	0.242
Duration of EH (months)	4 (0—10)	4 (1—10)	0.834
Diabetes mellitus [n (%)]	14 (16.09%)	20 (21.51%)	0.354
BMI (kg/m^2^)	24.95 ± 2.52	24.25 ± 2.86	0.082
SBP (mmHg)	151.71 ± 12.85	151.91 ± 12.30	0.944
DBP (mmHg)	85.01 ± 12.22	85.24 ± 12.33	0.897
Heart rate (bp)	71.10 ± 9.22	72.50 ± 9.97	0.314
TBil (μmol/L)	14.82 ± 6.34	14.7 ± 6.85	0.914
ALT (U/L)	24 (18—35)	24 (18—33)	0.913
LDH (U/L)	179.67 ± 56.28	180.34 ± 53.71	0.936
Uric acid (μmol/L)	366.00 ± 86.66	359.32 ± 102.74	0.640
eGFR (mL/min/1.73m^2^)	84.98 ± 16.15	84.72 ± 17.49	0.918
Total cholesterol (mmol/L)	5.04 ± 0.98	4.81 ± 1.11	0.130
HDL-C (mmol/L)	1.38 ± 0.41	1.37 ± 0.39	0.886
LDL-C (mmol/L)	3.00 ± 0.93	2.82 ± 0.93	0.205
FSG (mmol/L)	5.79 ± 0.99	6.01 ± 1.53	0.243
CIMT (cm)	0.06 ± 0.01	0.06 ± 0.01	0.597
UACR (mg/g)	7.37 (3.92—14.88)	6.14 (2.92—12.85)	0.088
BP compliance [n (%)]	74 (85.06%)	82 (88.17%)	0.539
LVMI (g/m^2^)	85.14 ± 21.85	84.43 ± 24.83	0.871
ACEI/ARBs [n (%)]	51 (58.62%)	41 (44.09%)	0.049
β-blockers [n (%)]	17 (19.54%)	16 (17.20%)	0.686
Calcium channel blockers [n (%)]	43 (49.43%)	54 (58.06%)	0.245
Diuretics [n (%)]	23 (26.44%)	42 (45.16%)	0.009
Statin [n (%)]	47 (54.02%)	44 (47.31%)	0.368
FMD (%)	4.02 ± 2.08	3.65 ± 3.57	0.376

Data are expressed as means ± SD or median (25th–75th).

*EH* essential hypertension, *BMI* body mass index, *SBP* systolic blood pressure, *DBP* diastolic blood pressure, *TBil* total bilirubin, *ALT* alanine aminotransferase, *LDH* lactate dehydrogenase, *HDL-C* high-density lipoprotein-cholesterol, *LDL-C* low-density lipoprotein-cholesterol, *FSG* fasting serum glucose, *CIMT* carotid intima-media thickness, *UACR* urinary albumin to creatinine ratio, *LVMI* left ventricular mass index, *ACEI* angiotensin-converting enzyme inhibitor, *ARB* angiotensin receptor blocker, *FMD* flow-mediated dilation.

**Table 3:** Clinical characteristics of patients with initial normal endothelial function who improved vs. those who did not improve after 3 months of enrollment treatment.

Variables	Improved group (n = 110)	Unimproved group (n = 166)	*P* value
Female, n (%)	44 (40.00%)	84 (50.60%)	0.084
Age (years)	55.22 ± 11.91	63.35 ± 11.01	< 0.001
Current smoking [n (%)]	19 (17.27%)	45 (27.11%)	0.058
Drinking [n (%)]	8 (7.27%)	22 (13.25%)	0.172
Duration of EH (months)	1 (0- 7)	2 (0- 10)	0.250
Diabetes mellitus [n (%)]	9 (8.65%)	15 (9.32%)	1.000
BMI (kg/m^2^)	23.99 ± 23.99	24.05 ± 2.94	0.876
SBP (mmHg)	151.0 ± 10.23	150.18 ± 10.03	0.487
DBP (mmHg)	86.66 ± 12.72	83.9 ± 11.23	0.059
Heart rate (bp)	72.64 ± 8.3	72.36 ± 8.79	0.795
TBil (μmol/L)	14.33 ± 6.84	14.0 ± 5.42	0.745
ALT (U/L)	23 (17—32)	23 (17—33)	0.876
LDH (U/L)	171.53 ± 38.12	176.89 ± 37.19	0.263
Uric acid (μmol/L)	332.96 ± 84.43	346.2 ± 84.08	0.222
eGFR (mL/min/1.73m^2^)	96.44 ± 14.52	87.29 ± 14.32	< 0.001
Total cholesterol (mmol/L)	4.86 ± 1.08	5.13 ± 1.15	0.063
HDL-C (mmol/L)	1.39 ± 0.40	1.36 ± 0.39	0.468
LDL-C (mmol/L)	2.98 ± 0.82	3.19 ± 1.07	0.098
FSG (mmol/L)	5.66 ± 1.07	5.7 ± 1.28	0.784
CIMT (cm)	0.06 ± 0.01	0.06 ± 0.01	0.352
UACR (mg/g)	7.44 (3.99—13.90)	7.47 (4.18—12.23)	0.846
BP compliance [n (%)]	102 (92.73%)	152 (91.57%)	0.727
LVMI (g/m^2^)	79.48 ± 17.92	80.19 ± 16.53	0.763
ACEI/ARBs [n (%)]	59 (53.64%)	86 (51.81%)	0.766
β-blockers [n (%)]	29 (26.36%)	54 (32.53%)	0.274
Calcium channel blockers [n (%)]	53 (48.18%)	85 (51.20%)	0.623
Diuretics [n (%)]	31 (28.18%)	49 (29.52%)	0.811
Statin [n (%)]	38 (34.55%)	40 (24.10%)	0.059
FMD (%)	14.16 ± 6.9	12.37 ± 4.37	0.017

Data are expressed as means ± SD or median (25th–75th).

*EH* essential hypertension, *BMI* body mass index, *SBP* systolic blood pressure, *DBP* diastolic blood pressure, *TBil* total bilirubin, *ALT* alanine aminotransferase, *LDH* lactate dehydrogenase, *HDL-C* high-density lipoprotein-cholesterol, *LDL-C* low-density lipoprotein-cholesterol, *FSG* fasting serum glucose, *CIMT* carotid intima-media thickness, *UACR* urinary albumin to creatinine ratio, *LVMI* left ventricular mass index, *ACEI* angiotensin-converting enzyme inhibitor, *ARB* angiotensin receptor blocker, *FMD* flow-mediated dilation.

### Relationship between EEFI and the incidence of STOD in EH patients

**Figure 2:**
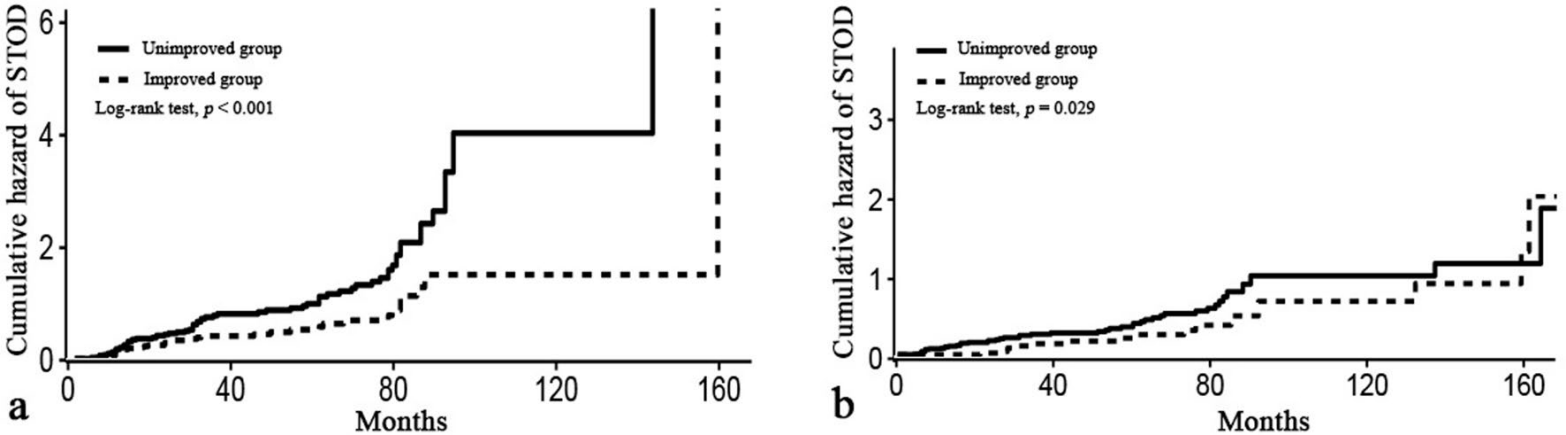
Cumulative hazard curves for STOD in the improved (dotted line) and unimproved (solid line) groups of EH patients with initial impaired (**a**) and unimpaired (**b**) endothelial function. *EH* essential hypertension, *STOD* subclinical target organ damage.

Kaplan–Meier survival curve analysis revealed a significantly higher incidence of STOD in EH patients with endothelial dysfunction than in those with normal function (Log-rank test, *p* < 0.001), as shown in Fig. S1 (Supplementary Fig. 1). Subsequent subgroup analysis with a cumulative hazard curve indicated that the group with improved endothelial function exhibited a significantly lower STOD incidence than the unimproved group (Log-rank test, *p* < 0.05) (Fig. 2).

### Cox regression analysis of the incidence of STOD in EH patients

**Figure 3:**
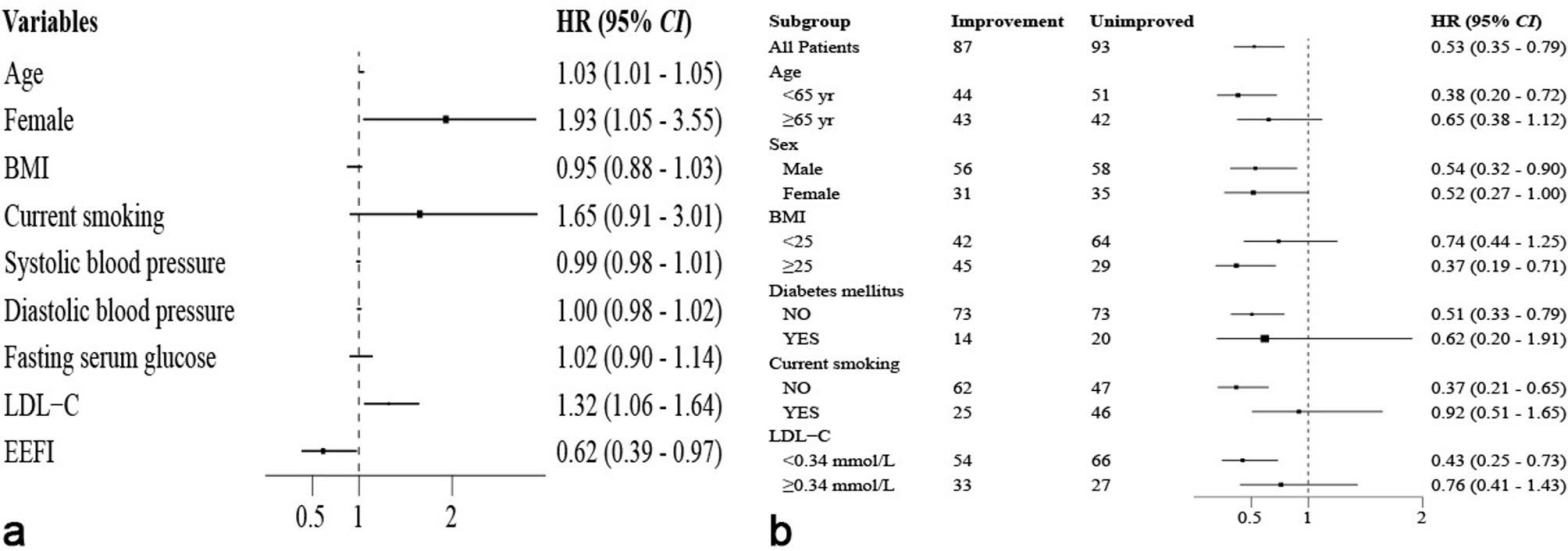
Cox regression analysis of STOD in patients with essential hypertension who had initial impaired endothelial function (**a**) and its subgroups (**b**). *STOD* subclinical target organ damage, *BMI* body mass index, *LDL-C* low-density lipoprotein-cholesterol, *EEFI* early endothelial function improvement.

**Figure 4:**
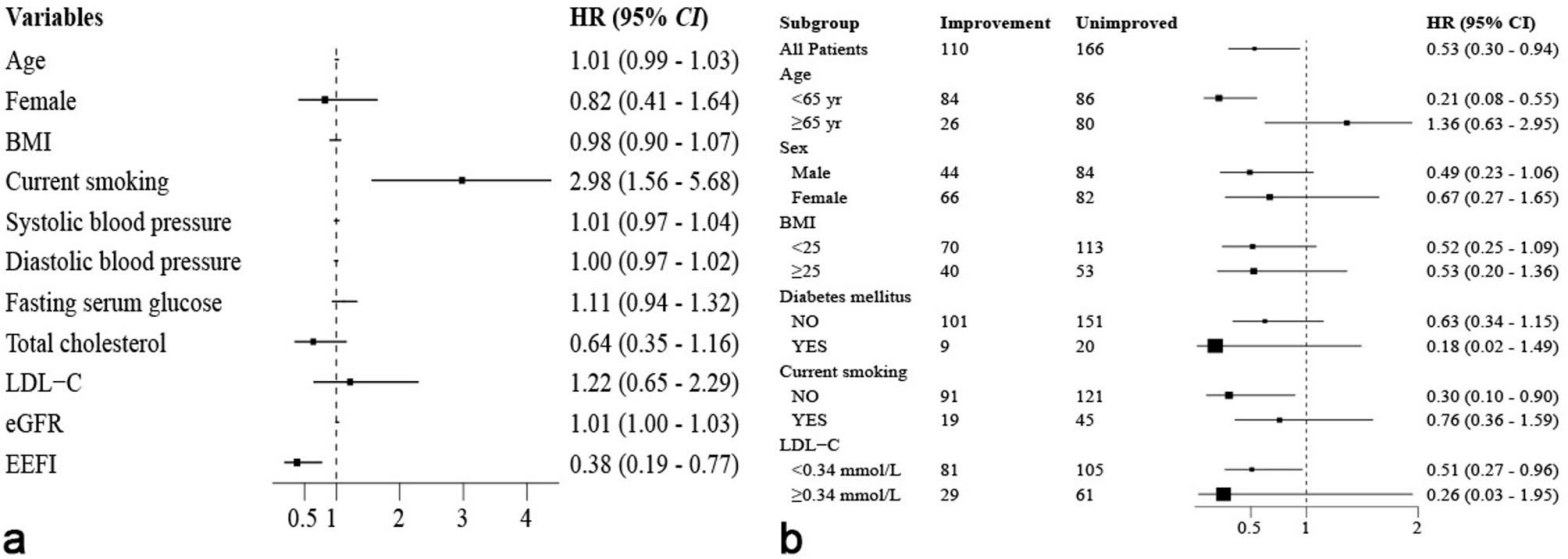
Cox regression analysis of STOD in patients with essential hypertension who had initial normal endothelial function (**a**) and its subgroups (**b**). *STOD* subclinical target organ damage, *BMI* body mass index, *LDL-C* low-density lipoprotein-cholesterol, *eGFR* estimated glomerular filtration rate, *EEFI* early endothelial function improvement.

Multivariate Cox regression analysis was conducted with EEFI, factors with significant differences or trends in the univariate analysis, and traditional risk factors (age, gender, BMI, smoking, blood pressure, FSG, and LDL-C) as independent variables to examine their association with STOD incidence. In patients with initial endothelial impairment, age, female and LDL-C emerged as independent risk factors for STOD, whereas EEFI (HR = 0.53, 95% CI 0.35–0.79) served as a protective factor (Fig. 3a). Subgroup analysis showed that EEFI significantly reduced the risk of STOD in patients under 65, male, overweight, non-diabetic, non-smokers, with LDL-C below 3.4 mmol/L. Conversely, its impact was not significant in patients 65 or older, female gender, not overweight, diabetic, smokers, or with LDL-C at or above 3.4 mmol/L (Fig. 3b). As illustrated in Fig. 4a, for patients with normal endothelial function, EEFI (HR = 0.53, 95% CI 0.30–0.94) continued to act as a protective factor against STOD, whereas smoking was the sole independent risk factor. Notably, the subgroup analysis revealed similar results to those in patients with initially impaired endothelial function, except for gender, BMI, and diabetes, which were not yet statistically different, as shown in Fig. 4b.

## Discussion

This retrospective cohort study analyzed 456 patients with EH over 16 years to evaluate the impact of EEFI on preventing STOD. Results showed that EH patients with endothelial dysfunction faced a significantly higher risk of developing STOD than those with normal function. More importantly, early improvement in endothelial function significantly lowered the cumulative incidence of STOD, particularly when blood pressure was maintained at target levels. Interestingly, the protective benefits of endothelial function improvement were more pronounced in certain groups, including patients under 65 years of age, nonsmokers, and with lower LDL-C levels.

Endothelial cells are crucial for vascular health, regulating vasodilation, vasoconstriction, anti-inflammatory responses, and anticoagulant functions^17^. In EH, endothelial dysfunction significantly increases the risk of developing STOD^18,19^. FMD is a well-established, accurate, and non-invasive indicator of peripheral endothelial function^6–10,13^ and is widely utilized in clinical research. Sun et al.^19^ found that adverse changes in FMD correlated with increases in LVH, UACR, and CIMT in a large cohort of EH patients. Furthermore, Yang et al.^20^ in a prospective study involving 199 EH patients, demonstrated that FMD was independently linked to the occurrence of STOD. This cohort study reaffirmed that EH patients who initially had endothelial dysfunction were much more likely to develop STOD compared to those with normal function. Mechanistically, endothelial dysfunction impairs nitric oxide (NO) production, leading to reduced vasodilation and increased vascular resistance, contributing to the advancement of hypertension^4,17^. Additionally, endothelial dysfunction is associated with increased oxidative stress and inflammation, further damaging vascular tissues and exacerbating blood pressure control^18^. Therefore, endothelial dysfunction is not only an independent predictor of STOD in EH patients but also a crucial target for clinical intervention.

This study showed that EEFI significantly reduced the cumulative incidence of STOD in EH patients, irrespective of their initial endothelial function. Studies have suggested that identifying and treating endothelial dysfunction can slow the progression of hypertension and decrease the risk of severe cardiovascular events^19,21^. Intervention studies have revealed that RAS inhibitors and statins not only provide foundational treatment but also enhance endothelial cell function, increase NO production, and improve vascular health^22–24^. After three months of consistent antihypertensive treatment, only a subset of patients experienced improvement in endothelial function, potentially due to the endothelial protective effects of RAS inhibitors and statins. Recent research has indicated that SIRT6, through epigenetic modulation, can prevent hypertension-induced endothelial dysfunction^21^. Non-pharmacological interventions, such as regular aerobic exercise^25,26^, dietary changes (e.g., mediterranean diet^27^, omega-3 fatty acids^28^), stress reduction techniques (e.g., yoga^29^), and lifestyle modifications like smoking cessation^30^, have been shown to improve endothelial function and promote vascular health in recent studies. However, reports on the early enhancement of endothelial function to reduce STOD in EH patients remain scarce. This study enhances our understanding that EEFI can serve as a preventive strategy to lower the risk of STOD in hypertensives.

This study also emphasized the importance of EEFI in specific populations. In hypertensive patients under 65 years, EEFI effects are more pronounced due to their greater vascular elasticity and stronger cellular regenerative capacity^31^. Male patients' positive response to EEFI may be due to their unique physiological and hormonal conditions. Research indicates that androgens may promote vasodilation in hypertensives by increasing the bioavailability of NO^32^. Overweight patients face a heightened risk of endothelial dysfunction, driven by elevated inflammation^33^. In this population, early intervention in endothelial function may reduce cardiovascular risks^34^. Hypertensive non-smokers typically exhibit lower oxidative stress^35^. Thus, they are better positioned to benefit from EEFI and show a stronger capacity for endothelial function recovery. Additionally, lower LDL-C and normal blood glucose support a healthy metabolic state, enhancing the endothelial cells' response to treatment and thereby promoting vascular health^36,37^. These findings underscore the need for targeted endothelial assessment and intervention in specific hypertensive subgroups to lower STOD risk and improve cardiovascular health, facilitating personalized treatment strategies.

However, this study had several limitations. The retrospective cohort design might have introduced information bias and missing variables, which were addressed through Cox regression analysis. Categorizing patients by endothelial function improvement within three months did not consider the effects of different medications or lifestyle changes. The single-center setting with a limited sample size, especially with few patients having initial endothelial dysfunction, may constrain the broader applicability. Importantly, we did not analyze major adverse cardiovascular events, which are essential for understanding long-term cardiovascular risks. We also did not measure endothelial glycocalyx biomarkers, which could have provided deeper insights into endothelial health. Although FMD is the gold standard for assessing endothelial function, it is time-consuming, expensive, and requires specialized equipment and personnel. Alternative methods like reactive hyperemia index, pulse wave velocity, and serum biomarkers (e.g., endothelin-1 and C-reactive protein) could be more practical^38^. Lastly, our study included only Asian patients, limiting our findings' applicability to other racial and geographic populations. Future research should include a more diverse patient population to validate and broaden these findings.

## Conclusion

In summary, this study demonstrated that meeting blood pressure targets in EH patients, combined with early enhancement of endothelial function, significantly decreased the risk of subsequent STOD. The results provide clinicians essential insights into assessing and intervening in endothelial function, recommending their incorporation into standard EH management protocols, particularly for patients under 65 years old, non-smoking, and with LDL levels below 3.4 mmol/L.

### Supplementary Information

Supplementary Figure 1.

## Data Availability

The datasets generated and analyzed during the current study are not publicly available due to privacy or ethical restrictions but are available from the corresponding author on reasonable request. The data were drawn from the *Hypertension Target Organ Damage and Its Risk Factors—Fuzhou Study* database.
