# Supplementary material for: Effect of early endothelial function improvement on subclinical target organ damage in hypertensives
Source: Sci Rep. 2024 Jul 12;14:16078. doi: 10.1038/s41598-024-67143-1 (PMC11239846; doi:10.1038/s41598-024-67143-1)
Supplement: Supplementary file 1 — Supplementary Figure 1. [file 41598_2024_67143_MOESM1_ESM.docx]

**
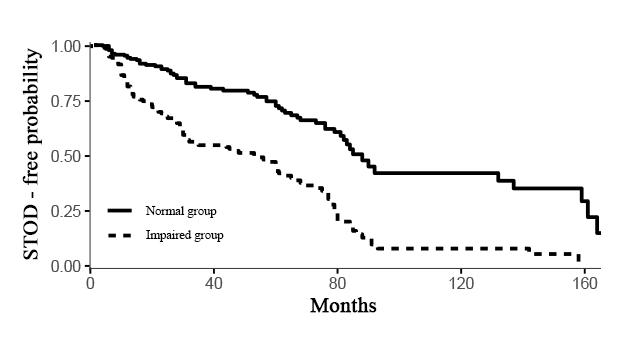
**

**Supplementary Figure 1.** Kaplan-Meier curves of STOD in EH patients with initially impaired (dotted line) and normal (solid line) function (Log-rank test, *p* < 0.001). EH: essential hypertension; STOD: subclinical target organ damage.
